# Single-nucleus chromatin landscapes during zebrafish early embryogenesis

**DOI:** 10.1038/s41597-023-02373-y

**Published:** 2023-07-19

**Authors:** Xiumei Lin, Xueqian Yang, Chuan Chen, Wen Ma, Yiqi Wang, Xuerong Li, Kaichen Zhao, Qiuting Deng, Weimin Feng, Yuting Ma, Hui Wang, Lveming Zhu, Sunil Kumar Sahu, Fengzhen Chen, Xiuqing Zhang, Zhiqiang Dong, Chuanyu Liu, Longqi Liu, Chang Liu

**Affiliations:** 1 College of Life Sciences, University of Chinese Academy of Sciences, Beijing, 100049 China; 2 BGI Research, Shenzhen, 518083 China; 3 BGI Research, Hangzhou, 310030 China; 4 Center for Neurological Disease Research, Taihe Hospital, Hubei University of Medicine, Shiyan, 442000 Hubei China; 5 College of Biomedicine and Health, College of Life Science and Technology, Huazhong Agricultural University, Wuhan, Hubei 430070 China; 6 BGI Research, Beijing, 102601 China; 7 China National GeneBank, Shenzhen, Guangdong 518120 China; 8 Shenzhen Bay Laboratory, Shenzhen, 518000 China

**Keywords:** Embryogenesis, Differentiation, Databases

## Abstract

Vertebrate embryogenesis is a remarkable process, during which numerous cell types of different lineages arise within a short time frame. An overwhelming challenge to understand this process is the lack of dynamic chromatin accessibility information to correlate *cis*-regulatory elements (CREs) and gene expression within the hierarchy of cell fate decisions. Here, we employed single-nucleus ATAC-seq to generate a chromatin accessibility dataset on the first day of zebrafish embryogenesis, including 3.3 hpf, 5.25 hpf, 6 hpf, 10 hpf, 12 hpf, 18 hpf and 24 hpf, obtained 51,620 high-quality nuclei and 23 clusters. Furthermore, by integrating snATAC-seq data with single-cell RNA-seq data, we described the dynamics of chromatin accessibility and gene expression across developmental time points, which validates the accuracy of the chromatin landscape data. Together, our data could serve as a fundamental resource for revealing the epigenetic regulatory mechanisms of zebrafish embryogenesis.

## Background & Summary

Understanding embryonic development in vertebrates can not only shed light on how it evolved and how various species are related, but also help in resolving critical questions related to devastating diseases such as tumors and degenerative diseases. It is crucial to explore the genetic and epigenetic mechanisms behind cell lineage differentiation and tissue formation during vertebrate embryonic development^1,2^. Zebrafish is an ideal model organism for studying vertebrate embryonic development owing to its quick development and versatility for manipulation^3^, and efforts have been made to systematically describe the key events and characteristics from the 1-cell to organogenesis stage, which provides valuable resources for the follow-up study of molecular regulatory mechanisms at different developmental stages^4^.

The coordination of chromatin structure and transcription is essential to gene regulation and cell fate determination in embryonic development^5^. During early embryogenesis, there exists an asynchronism between DNA accessibility of *cis*-regulatory elements (CREs) and gene expression, which has been proven that DNA accessibility within CERs precedes expression of target genes^6,7^. Additionally, chromatin architecture undergoes dramatic changes through early embryonic development. Before zygotic genome activation (ZGA), cells are in a stage of rapid proliferation, and the formation of extensive higher-order chromatin structure is inhibited. Accompanied by the slowing down of cell divisions and introduction of cell cycle gap phases, the higher-order chromatin structure is initially established post-ZGA^8^. This establishment, which is the basis for interaction-regulation between CREs including enhancers and promoters, is important for gene regulatory networks (GRNs) during embryonic development. Efforts have been made to map the genomic regulatory landscapes in bulk^9,10^ and at single-cell resolution within a single development stage in zebrafish^11^, but the dynamic chromatin landscapes of the single-nucleus data during zebrafish early embryogenesis are still obscure, which could systematically delineate the dynamics of CREs and its synergistic interaction with gene expression to regulate cell fate differentiation.

In the present study, we adopted single-nucleus assay for transposase-accessible chromatin with high-throughput sequencing (snATAC-seq) at seven different time points during the first day of zebrafish embryo development, generating accessibility profiles for 51,620 nuclei, and establishing a resource for dynamics of CREs during zebrafish early embryogenesis. Furthermore, through the integration of snATAC-seq data and single-cell RNA sequencing (scRNA-seq) data at the same developmental time point, we systematically characterized the dynamics of chromatin accessibility and gene expression in different cell types across the examined developmental time points, which exhibited a good congruence between these two datasets. Our study constitutes a fundamental reference for further studies aiming to unravel the complex GRNs of early vertebrate embryonic development.

## Materials and Methods

### Experimental animal and samplings

The animals used in this study were approved by the Animal Care and Use Committee of Huazhong Agriculture University (HZAUFI-2021-0001). Zebrafish embryos from AB wild-type crosses were collected at 3.3 hours, 5.25 hours, 6 hours, 10 hours, 12 hours, 18 hours, and 24 hours post fertilization (hpf). For snATAC-seq, the 250–1,000 embryos from different developmental stages were collected in a 2 mL centrifuge tube (AXYGEN, 28820394) and performed liquid nitrogen snap-frozen immediately after cleaning the tissue fluid with 1x phosphate-buffered saline (PBS) solution (Meilunbio, MA0015). The 6 hpf embryos acquired in this study were processed for scRNA-seq as described before^12^.

### Nuclei isolation for snATAC-seq

Nuclei were isolated from zebrafish embryos for snATAC-seq according to the mechanical extraction method with slight modification^13^. In brief, we thawed the embryos that were snap-frozen in liquid nitrogen and added 2 mL pre-chilled homogenization buffer (Sigma, CELLYTPN1-1KT) into the tube. Each 1 mL mixture was transferred to a 2 mL Dounce homogenizer (Kimble, NO.885300-0002) on ice, and then homogenized with 10–20 strokes of the tight pestle (the B pestle). Next, the homogenized mixture was filtered into a 1.5 mL tube through a 40 µm cell strainer (Falcon, 352340), followed by centrifugation at 1,200 g for 5 minutes at 4 °C to pellet the nuclei, and then suspended in a blocking buffer containing 1% bovine serum albumin (BSA) and 0.2 U/μL RNase inhibitor in 1 × PBS. Finally, the nuclei were pelleted for a second time by centrifugation at 500 g for 5 minutes at 4 °C, and resuspended in 1 × PBS containing 1% BSA for following libraries preparation.

### snATAC-seq library construction and sequencing

snATAC-seq libraries were prepared using DNBelab C Series Single-Cell ATAC Library Prep Set (MGI, 1000021878). Briefly, barcoded snATAC-seq libraries were constructed following a series of steps including transposition, droplet encapsulation, pre-amplification, emulsion breakage, capture beads collection, DNA amplification and purification. The libraries quality control was performed by using Agilent Bioanalyzer 2100 (Agilent Technologies, G2939A) and Qubit ssDNA Assay Kits (Thermo Fisher Scientific, Q10212). The paired-end sequencing was performed on MGI DNBSEQ-T7 using the following read length: 109 bp for read 1, 50 bp for read 2, and 10 bp for the sample index.

### Single cell isolation for scRNA-seq

The isolation of single cell suspension was performed as previously described^12^. Briefly, 250–1,000 embryos at 6 hpf were collected and the eggshells were digested using Pronase E (Sigma-Aldrich, P5147-1G). Once achieving a hatch rate of 20–30%, add 1 mL pre-warmed (56°C) high fetal calf serum (Hi-FBS, Biological Industries, 04-001-1ACS) to the dish to halt the reaction. The embryos were subsequently washed once with 0.5x Danieau’s solution supplemented with 10% Hi-FBS, followed by thrice with 0.5x Danieau’s solution. Then the embryos were treated with deyolking buffer and digested the embryos with 1 × trypsin-EDTA solution (Biosharp, BL512A) to obtain the single-cell suspension. Finally, the isolated single cells were resuspended in 1x PBS (Meilunbio, MA0015) supplemented with 0.04% BSA (Sigma, A8022-50G) for subsequent libraries construction.

### scRNA-seq library construction and sequencing

The scRNA-seq libraries of 6 hpf acquired in this study were prepared using the DNBelab C Series High-throughput Single-Cell Library Preparation Kit (MGI, 940-000047-00) according to the manufacture’s protocol and previous report^12^. In brief, after droplet generation, emulsion breakage, reverse transcription and cDNA amplification were performed to generate the sRNA-seq libraries. Finaly, the libraries were finally sequenced on an MGI DNBSEQ-T7 using the paired-end strategy: 41 bp for read 1, 100 bp for read 2, and 10 bp for the sample index.

### Bioinformatics preprocessing

#### snATAC-seq data processing

The raw reads were processed by PISA (version 1.1), and then aligned to the zebrafish genome (GRCz11) by BWA (version 0.7.17-r1188) to generate the BAM files. Then bap2^14^ was used to create fragment files for each snATAC-seq library. Finally, the fragments larger than the chromosome length and those smaller than 50 bp were filtered out to generate clean fragments for subsequent analysis by ArchR (version 1.0.2).

#### scRNA-seq data processing and unsupervised clustering

Raw reads of 6 hpf data were processed using DNBelab_C_Series_HT_scRNA-analysis-software (https://github.com/MGI-tech-bioinformatics/DNBelab_C_Series_HT_scRNA-analysis-software) which includes alignment, primary filtering, and gene expression matrix generation of each cell. We created a Seurat object by Seurat (V4.0.3) and filtered the low-quality cells according to gene number as well as the percentage of mitochondrial genes of each cell (nFeature_RNA > 800, nCount_RNA > 1,000 and percent.mt < 5). Then we merged the scRNA-seq data of 6 hpf with the previously published scRNA-seq data of other developmental stages^12^ for unsupervised clustering. The normalization and scaling were performed using Seurat ‘NormalizeData’ and ‘ScaleData’ function with the default parameters. Next, we performed dimension reduction by principal component analysis (PCA) and clustering by using ‘FindNeighbors’ and ‘FindClusters’ function. Finally, the differential expressed genes (DEGs) of each cluster were identified through the ‘FindAllMarkers’ function with default parameters.

#### Quality control, dimensionality reduction and clustering of snATAC-seq data

We created ArchR projects at different development stages using ‘ArchRProject’ function, and performed a Tn5 insertion correction with a positive chain offset of +4 bp as well as a negative chain offset of - 5 bp. Low-quality nuclei were filtered out based on unique nuclear fragments (nFrags) and enrichment score of transcriptional start site (TSS) with the following filtering criteria: log10 (nFrags) ≥ 3.8 and TSS score ≥ 4 for 3.3 hpf & 5.25 hpf; log10 (nFrags) ≥ 3.5 and TSS score ≥ 4 for 6 hpf; log10 (nFrags) ≥ 3.5 and TSS score ≥ 5 for 10 hpf; log10 (nFrags) ≥ 3.6 and TSS score ≥ 5 for 12 hpf; log10 (nFrags) ≥ 3.6 and TSS score ≥ 4 for 18 hpf, log10 (nFrags) ≥ 3.4 and TSS score ≥ 5 for 24 hpf. We calculated the doublet score using ‘addDoubletScores’ function with the default parameters and doublets were filtered out using ‘filterDoublets’ function to obtain high quality nuclei for the following analysis (filterRatio = 5).

Dimensionality reduction was performed using iterative latent semantic indexing (LSI). The ‘addClusters’ function based on the Seurat Leiden clustering algorithm was adopted for unsupervised clustering and the Uniform Manifold Approximation and Projection (UMAP) (default parameters) was utilized for visualization. Gene activity scores were calculated by ‘addGeneScoreMatrix’ function (default parameters) and the marker genes for each cluster were identified by ‘getMarkerFeatures’ function (FDR ≤ 0.01 & Log2 FC ≥ 1). The cell types annotation was performed using known cell-type–specific markers for each cluster and the marker genes were visualized by Integrative Genomic Viewer (IGV)^15^ (promoter +/− 2 kb).

#### Integration of snATAC-seq data and scRNA-seq data

We used the ‘addGeneIntegrationMatrix’ function to integrate snATAC-seq data and scRNA-seq data in ArchR. Briefly, CCA algorithm was utilized to calculate the correlation between gene expression matrix of scRNA-seq data and gene score matrix of snATAC-seq data with the shared features, and then we used ‘TransferData’ and ‘AddMetaData’ function to assign the label of scRNA-seq cell type to the snATAC-seq cell type based on prediction scores. To assess the similarity between the snATAC-seq and scRNA-seq datasets, we calculated the proportion of cells in the snATAC-seq cell type that shared labels with cells in the scRNA-seq cell type.

#### Peak-Gene link calculation

We performed the correlation analysis between gene expression and peak to identify peak-gene links by using ‘addPeak2GeneLinks’ function in ArchR and drew peak-gene links heatmap using ‘plotPeak2GeneHeatmap’ function with a correlation greater than 0.75.

#### Peak calling

We used the ‘addGroupCoverages’ function to create pseudo-bulk replicates for each cell type/developmental stage and performed the peak calling by using ‘addReproduciblePeakSet’ function with the following parameters: groupBy = “Clusters”, pathToMACS = pathToMacs2, “–nomodel”, genomeSize = 1345118429 for cell type; groupBy = “Stage”, pathToMACS = pathToMacs2, “–nomodel”, genomeSize = 1345118429, minCells = 3500, and maxCells = 20000 for developmental stage.

#### Motif database construction

We download the motif PWM matrix and the motif information of zebrafish from CIS-BP database (http://cisbp.ccbr.utoronto.ca/index.php) and used TFBSTOOLS (version 1.34.0) to convert PWM matrices into PWMatrixList objects. Then we annotated the peaks using ‘addMotifAnnotations’ function in ArchR with the parameters: motifset = Null, motifPWMs = PWMMatrixList.

#### Motif enrichment analysis

We performed cell-type differential peak analysis using ‘getMarkerFeatures’ function (FDR < 0.05 & Log2 FC > 1). Motif enrichment was performed through the ‘peakAnnoEnrichment’ function. The top 10 cell-type-specific motifs with significant adjusted P value were displayed by heatmap, and the corresponding transcription factors (TFs) expression were represented by genescore in snATAC-seq data and unique molecular identifiers (UMIs) in scRNA-seq data respectively.

#### TF footprinting analysis

We utilized the ‘getPositions’ function with default parameters to extract the positions of the relevant motifs. Subsequently, we employed the ‘addGroupCoverages’ function and ‘getFootprints’ function to obtain cell-type specific TF footprints. Finally, the TF footprints were visualized using the ‘plotFootprints’ function.

## Data Records

All raw data (FASTQ file) generated in this study by snATAC-seq (3.3 hpf, 5.25 hpf, 6 hpf, 10 hpf, 12 hpf, 18 hpf and 24 hpf) and scRNA-seq (6 hpf) have been deposited to CNGB Nucleotide Sequence Archive (CNSA)^16^ of China National GeneBank DataBase (CNGBdb)^17^ with accession number: CNP0002827^18^ and the NCBI Sequence Read Archive with the BioProject accession: PRJNA987386^19^. Additional, the gene expression matrix and the metadata, as well as the fragments were also submitted to CNGB Nucleotide Sequence Archive respectively (https://db.cngb.org/search/project/CNP0002827/)^18^. The cell-type peak matrices had also uploaded to Figshare (https://figshare.com/articles/dataset/Supplementary_Data/23171099)^20^ as well. All of these various data formats had been compiled in the Supplementary Table 1 (available at Figshare^20^), which consists of descriptions of data types and corresponding download links to facilitate easy data access.

## Technical Validation

**Fig. 1:**
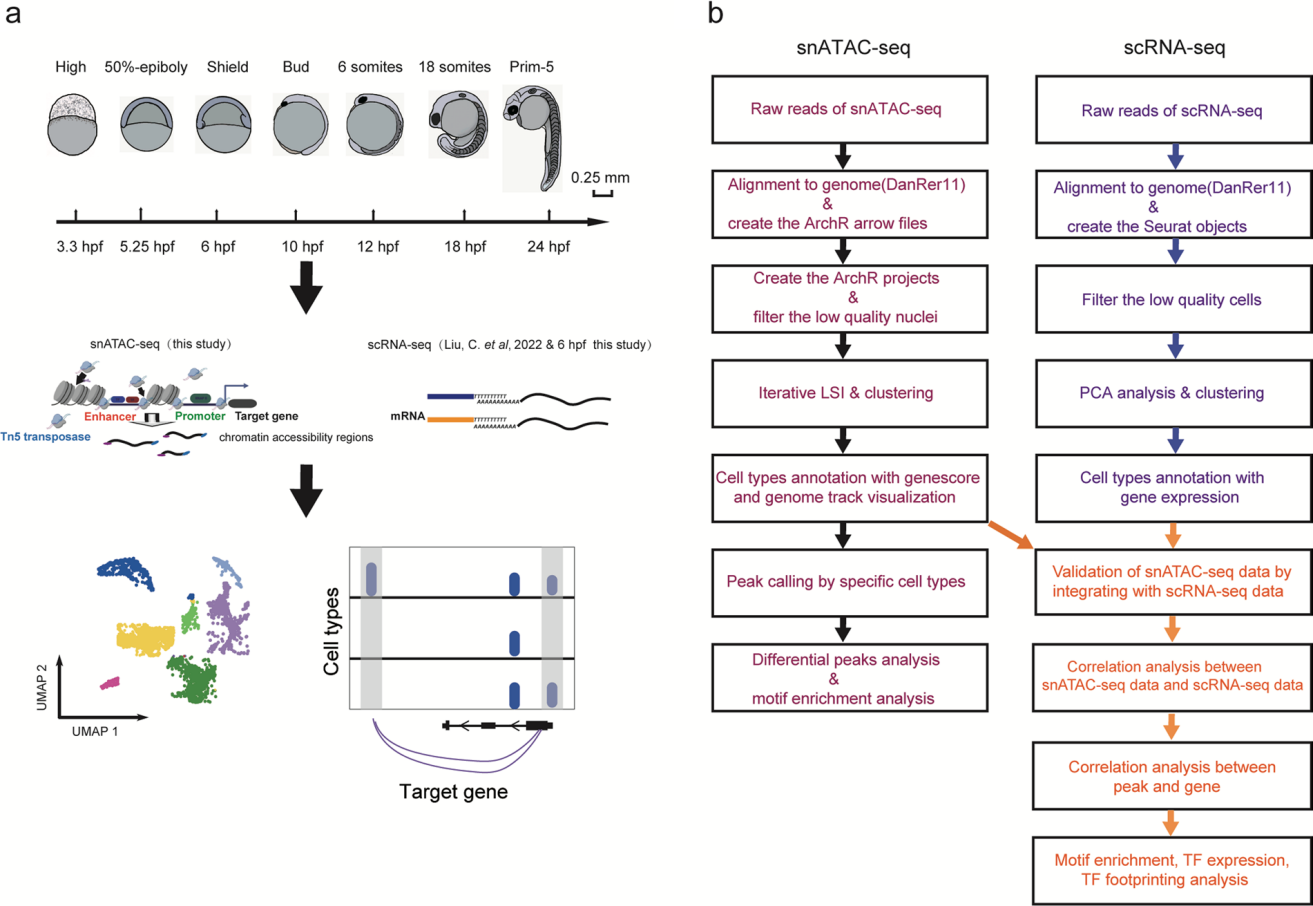
An overview of the experimental and data analysis workflow. (**a**) Experimental outline of the zebrafish embryos at different development stages (top) and snATAC-seq and scRNA-seq process diagram (bottom). Scale bar: 0.25 mm. (**b**) Analyses workflow for snATAC-seq and scRNA-seq profiles.

**Fig. 2:**
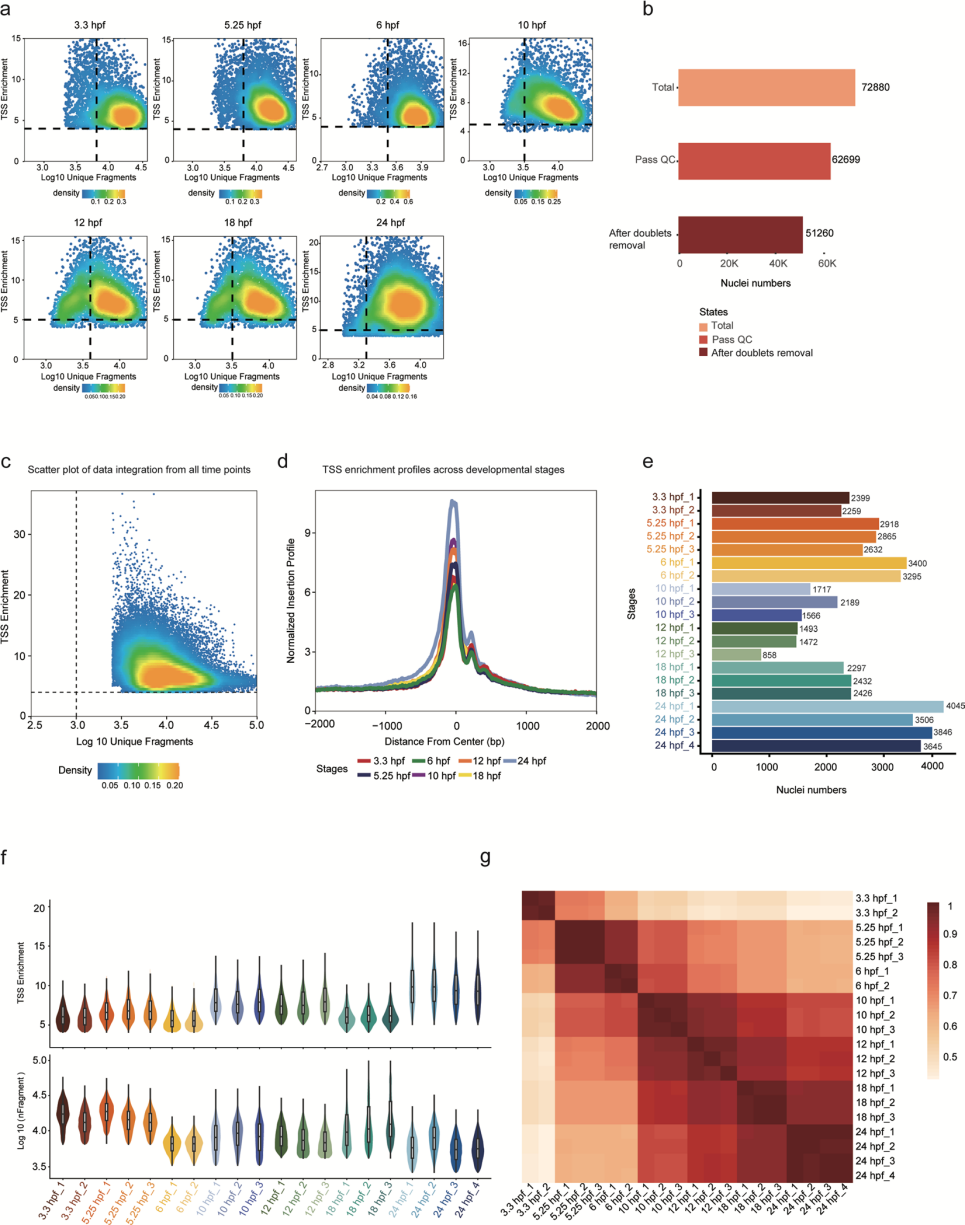
snATAC-seq data quality control and features. (**a**) Scatter plots showing bivariate distributions of TSS enrichment scores and log10 (unique fragments) of individual developmental time piont. (**b**) Histogram showing the nuclei numbers of raw processing data, data after filtering and data after doublet removal. (**c**) Scatter plots showing bivariate distributions of TSS enrichment scores and log10 (unique fragments) of data integration from all time points. (**d**) Plots showing the enrichment of snATAC-seq fragments around TSSs. (**e**) Histogram showing the nuclei numbers of all 20 snATAC-seq profiles. (**f**) Violin Plots showing the TSS enrichment scores (top) and unique fragment numbers (bottom) of all 20 snATAC-seq profiles. (**g**) Heatmap clustering of correlation coefficients across all 20 snATAC-seq profiles.

Zebrafish embryos from AB wild-type crosses were harvested for snATAC-seq at developmental stages of blastulation (3.3 hpf), gastrulation (5.25 hpf, 6 hpf, 10 hpf) and segmentation (12 hpf, 18 hpf, 24 hpf) (Fig. 1a). For the data reliability, we set up 2–4 biological replicates at each developmental stage for snATAC-seq library construction (Methods). The data analyses were processed via a standard pipeline (Fig. 1b). For quality control (QC), analyses were performed to filter low-quality data, in which the transcriptional start site (TSS) enrichment scores and unique fragments for each nucleus of each developmental stage were calculated (Fig. 2a). Here, as the scatter plot distribution varies across each developmental time point, the filtering criteria employed to remove nuclei with low fragments and TSS enrichment scores differ for each respective developmental time point. After QC and further doublet removal, a total of 62,699 and 51,620 high-quality nuclei were obtained for all time points (Fig. 2b, Table 1), where the TSS enrichment scores were mostly distributed between 5–10, and the numbers of unique fragments were mainly distributed between 5,000–20,000 (Fig. 2c, Table 1). Meanwhile, we identified 29,008–89,619 non-redundant peaks across all developmental stages respectively (Table 1) and observed a clear peak of fragments around the annotated TSSs (Fig. 2d). Furthermore, the nuclei numbers (Fig. 2e), TSS enrichment scores (Fig. 2f, top) and unique fragment numbers (Fig. 2f, bottom) of each replicate at each time point were overall generated for further analyses. Additionally, heatmap clustering of Pearson correlation coefficients from the comparison of 20 profiles presented a high correlation between replicates of the same developmental time point which indicated a high reproducibility of biological and technical replicates (Fig. 2g).

**Table 1:** An overview of QC parameters for the snATAC-seq profiles established in the developing zebrafish embryos.

Stages	3.3 hpf	5.25 hpf	6 hpf	10 hpf	12 hpf	18 hpf	24 hpf
Parameters							
Cell Number	4,658	8,415	6,695	5,472	3,823	7,155	15,042
Median Fragment	15,224	15,269	6,544	8,582	7,548	10,865	5,974
TSS Enrichment	6.00	6.68	5.56	7.76	7.56	6.14	9.61
FragsInPeaks(number)	10,606	13,707	5,543	8,550	7,338	9,108	5,541
FragsInPeaks (%)	69.76%	89.77%	84.71%	99.63%	97.22%	83.83%	92.75%
Peak Number	89,619	33,234	32,577	44,424	29,008	38,675	51,098

**Fig. 3:**
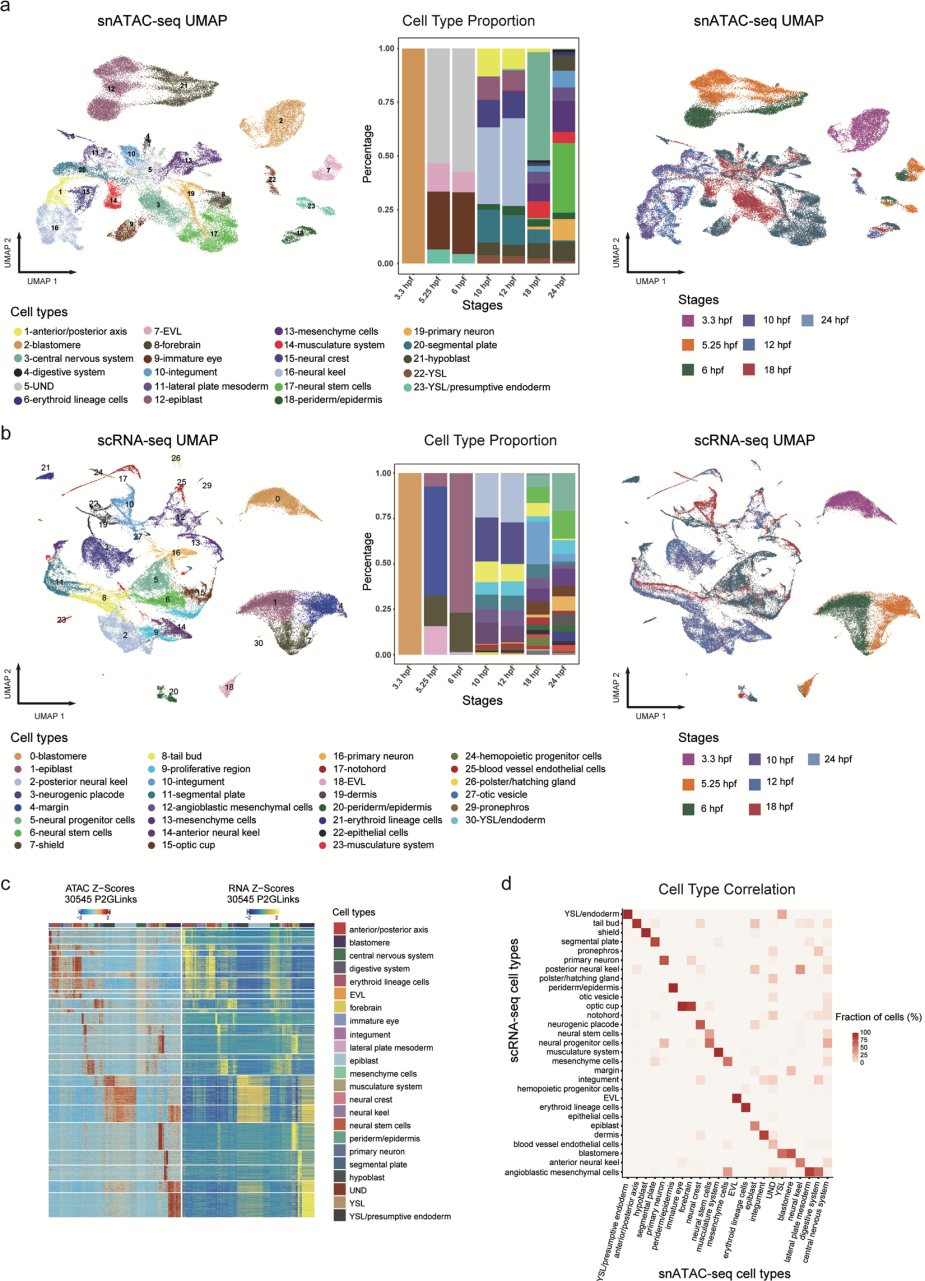
Clustering and annotation of the chromatin accessibility and gene expression patterns of the developing zebrafish embryos. (**a,b**) Clustering and UMAP visualization of snATAC-seq data (**a**) and scRNA-seq data (**b**) of all the seven developmental stages colored by cell types (left) and developmental stages (right). Histogram showing the percentage of specific cell type across the seven developmental stages (middle). The color legend of the left and middle panels is the same one. (**c**) Heatmap of peak-to-gene links in the developing zebrafish embryos generated using ArchR. (**d**) Heatmap showing the proportion of cells in the scRNA-seq clusters that overlaps with snATAC-seq defined clusters.

Subsequently, nuclei from all developmental time points were integrated and clustered by using ArchR (Methods) for cell-type-specific regulatory annotation. We calculated the gene activity scores by summing the fragments in the gene promoter and gene body to annotate cell clusters (Methods), and two of these clusters were excluded because of too few cells and the absence of cluster-specific genes. Finally, 23 clusters were identified as candidate cell types including the integumentary system (enveloping layer (EVL), periderm/epidermis, integument), nervous system (neural stem cells, forebrain, immature eye, neural keel), musculature system, digestive system, *et al*. (Fig. 3a, left). The marker genes for per cluster could be found in Supplementary Table 2 (available at Figshare^20^). By comparing the proportions of cell types at different time points (Fig. 3a, middle), we found a gradual increase in cell types along the development. Then, based on the UMAP visualization of snATAC-seq data and colored by developmental stages (Fig. 3a, right), we observed that the cells of blastulation, gastrulation and segmentation were discretely aggregated and separated from each other.

**Fig. 4:**
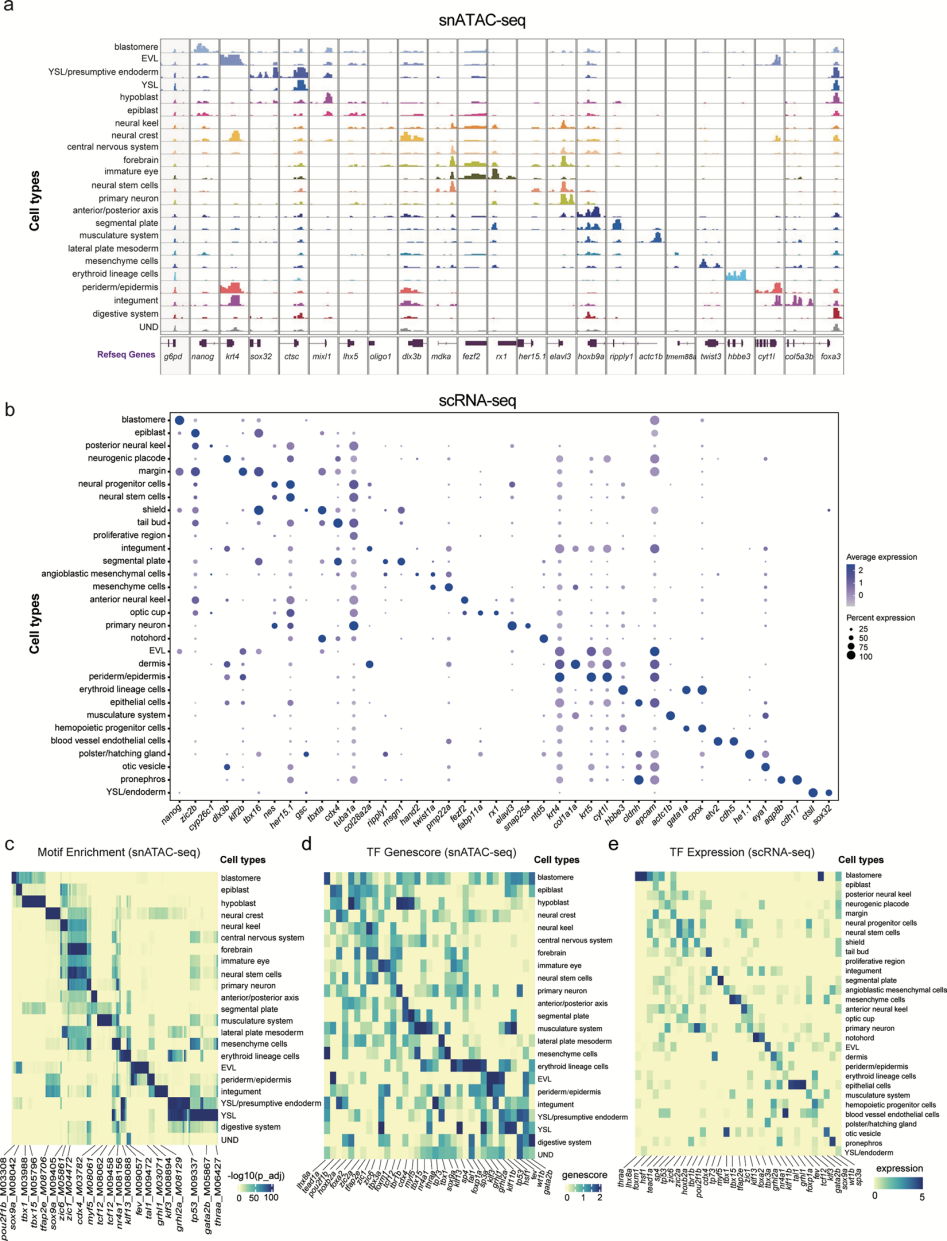
Combined analysis of snATAC-seq data and scRNA-seq data of the developing zebrafish embryos. (**a**) Aggregated chromatin accessibility profiles of each cell type at representative marker gene loci in snATAC-seq data and visualized by genome browser tracks (+/− 2 kb around the promoter). (**b**) A paired dot plot of scaled expression of representative marker genes in scRNA-seq data. (**c**–**e**) TF motifs enrichment (**c**), TFs activity gene score (**d**) and TFs gene expression (e) for different cell types.

**Fig. 5:**
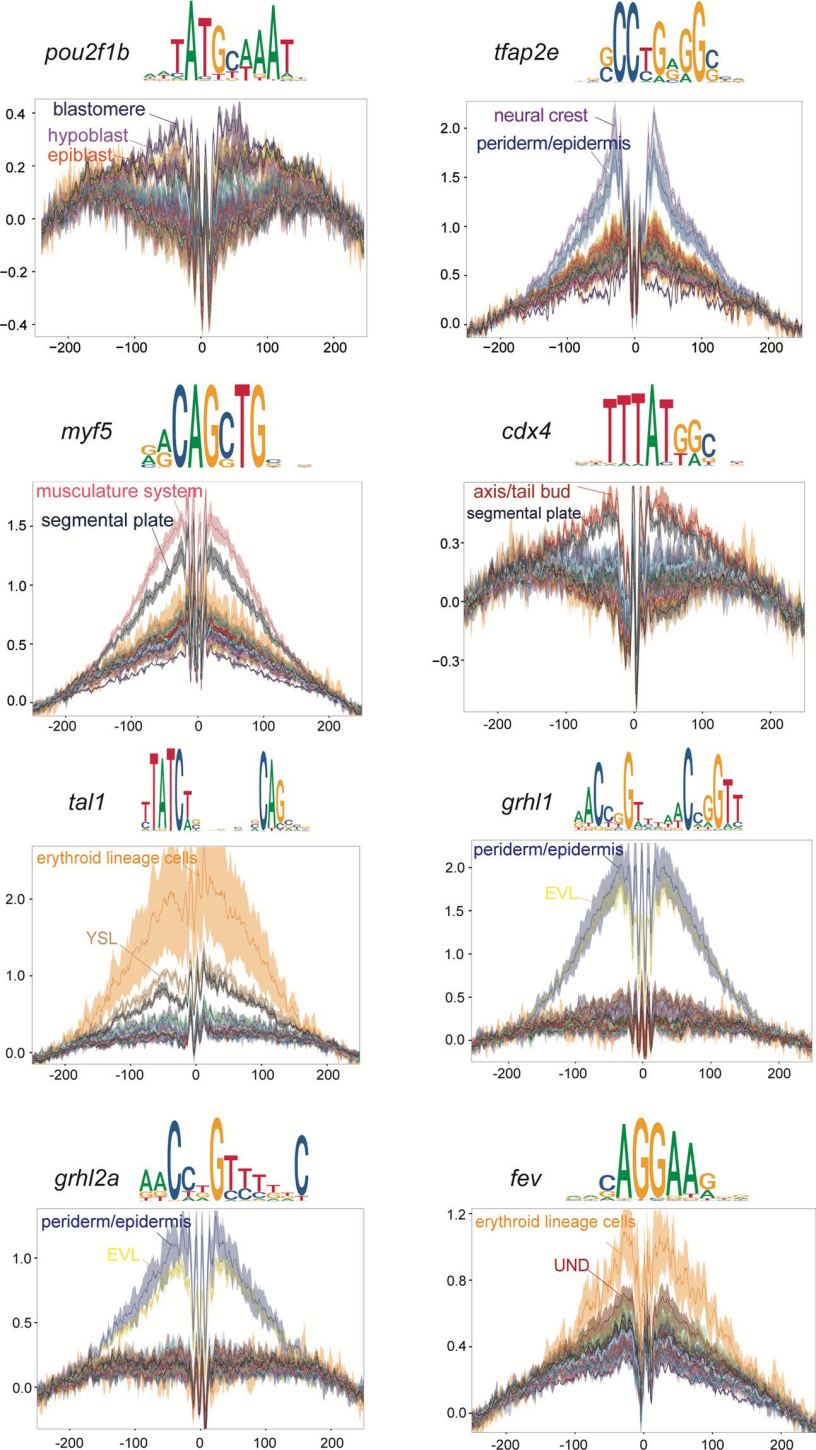
Footprint analysis identifies representative cell type specific TFs activities in snATAC-seq data. (top) Representative cell type specific TFs binding motif sequence logo, (bottom) representative cell type specific TFs footprint profiles. Related to Fig. 4.

In order to validate the accuracy of the chromatin landscape data, we inspected the consistency of the snATAC-seq data and the corresponding scRNA-seq data (Fig. 1a,b). We performed unsupervised clustering analysis on our previously published scRNA-seq dataset^12^ (including 3.3 hpf, 5.25 hpf, 10 hpf, 12 hpf, 18 hpf and 24 hpf) combined with unpublished scRNA-seq data of 6 hpf embryos acquired in this study and identified 30 clusters (the cluster 28 was excluded because of too few cells and the absence of cluster-specific genes) (Fig. 3b). The differential expression gene for per cluster could be found in Supplementary Table 3 (available at Figshare^20^). Furthermore, by integrating these two datasets, we observed a good correlation between gene activity scores of snATAC-seq data and gene expression values of scRNA-seq data (Fig. 3c), and a high congruence with membership between these two datasets (Fig. 3d). Meanwhile, we found that the genes with accessible elements around the promoter in the chromatin landscapes (Fig. 4a) also had a high expression level in the corresponding celltypes of scRNA-seq data (Fig. 4b), such as *nanog* which is involved in the maintenance of pluripotency at the blastula^21^, *sox32* which is especially expressed in the yolk syncytial layer (YSL)/endoderm and involved in endoderm formation^22^, and *elavl3* which is expressed specifically in primary neuron and involved in neurogenesis^23^. To further dissect the regulators of different cell types in the snATAC-seq data, we compared the transcription factors (TFs) motif enrichment (Fig. 4c) and TFs gene activity score (Fig. 4d) generated by the snATAC-seq data with TFs expression (Fig. 4e) obtained from the scRNA-seq data. These analyses exhibited a good congruence between them in the corresponding cell types, such as *myf5* in somite, *grhl1* in the EVL, *tal1* in the erythroid lineage cells, *cdx4* in the tail bud and anterior/posterior axis. In order to validate the identified motifs, we performed the TF footprinting analysis, which confirmed the binding of TFs to DNA. We found that the motifs exhibit active TF binding in the corresponding cell types (Fig. 5). In summary, we generated the chromatin accessibility profiles for zebrafish early embryogenesis, which proved a high concordance with the corresponding published scRNA-seq data^12^.

Taken together, our datasets provide a valuable resource for in-depth exploration of the epigenetic regulation mechanism during the zebrafish embryo development.

## Usage Notes

The pipeline of the snATAC-seq and scRNA-seq data processing, including the read mapping, low-quality cells filtering, unsupervised clustering and peak calling were run on the Linux operating system. All R/Python source codes with the optimized parameters used for the downstream data analyses and visualization are provided online.

## Data Availability

The codes used to analyze the data in this study were available online (https://figshare.com/articles/dataset/Code/22121171)^24^.
